# The 5-min Apgar score and primary school performance: a Dutch nationwide cohort study

**DOI:** 10.1007/s00431-025-06526-6

**Published:** 2025-10-25

**Authors:** Flo Anema, Anita C. J. Ravelli, Wes Onland, Petra C. A. M. Bakker, Floris Groenendaal, Ageeth N. Rosman, Jasper V. Been, Carline E. Tacke, on behalf of N3 Benchmarking group, T. A. J. Antonius, P. H. Dijk, K. P. Dijkman, S. Koole, R. F. Kornelisse, F. A. B. A. Schuerman, M. L. Tataranno, E. van Westering-Kroon, R. S. G. M. Witlox, Wes Onland, Floris Groenendaal, Carline E Tacke

**Affiliations:** 1 Department of Neonatology, Emma Children’s Hospital, Amsterdam University Medical Centers, Meibergdreef 9, 1105 AZ Amsterdam, the Netherlands; 2 Department of Obstetrics and Gynaecology, Amsterdam University Medical Centers, Amsterdam, the Netherlands; 3 Department of Medical Informatics, Amsterdam University Medical Centers, Amsterdam, the Netherlands; 4 Amsterdam Reproduction & Development Research Institute, Amsterdam, the Netherlands; 5 Department of Neonatology, Wilhelmina Children’s Hospital, University Medical Center Utrecht, Utrecht University, Utrecht, the Netherlands; 6 Perined, Utrecht, the Netherlands; 7 Department of Neonatal and Paediatric Intensive Care, Division of Neonatology, Erasmus MC Sophia Children’s Hospital, University Medical Centre Rotterdam, Rotterdam, the Netherlands; 8 Department of Obstetrics and Gynaecology, Erasmus MC Sophia Children’s Hospital, University Medical Centre Rotterdam, Rotterdam, the Netherlands

**Keywords:** Apgar score, Educational outcome, Childhood, Development

## Abstract

The purpose of this study is to investigate the association between the 5-min Apgar score and primary school performance. The national cohort study involves singletons born between 35+ 0–42 + 6 weeks of gestation from 2000 to 2009. The Dutch Perinatal Registry was linked with Statistics Netherland’s database to study educational outcomes at primary school, focusing on special education use and a high track recommendation for secondary school at age 12. Multivariate (adjusted) logistic regression assessed the association between Apgar score (grouped as 0–3, 4–6, 7, 8, 9, and 10) and these outcomes. Of the 1,618,087 children available, a total of 6.4% attended special education at primary school. The highest rate of 14.3% was observed in the group with the lowest Apgar scores (0–3), gradually decreasing to the lowest rate of 6.0% in the group with the highest Apgar score. Compared with a score of 10, the lowest scores had the highest odds ratio for use of special education (adjusted OR 2.4; 95% CI 2.1–2.8). Similarly, lower Apgar scores were associated with a reduced likelihood of receiving a high track recommendation for secondary school. The lowest rate of a high track recommendation (40.7%) was found in children with Apgar score of 0–3, increasing to 45.0% in those with score of 10. Even Apgar scores of 7, 8 and 9 showed significantly lower odds for, compared with a score of 10.

*Conclusion*: There is an association between the Apgar score and primary school performance, with lower Apgar scores linked to poorer educational outcomes.

**Supplementary Information:**

The online version contains supplementary material available at 10.1007/s00431-025-06526-6.

**Table:** 

**What is Known:** • *The 5-min Apgar score has been linked to educational performance, with poorer outcomes being reported in children with lower scores*. • *An increasing trend in low Apgar scores has been observed in the Netherlands*. **What is New:** • *There is an association between the full range of Apgar scores and primary school performance, with lower scores being linked to poorer educational performance*. • *Children born with Apgar score of 7, 8 or 9 had a significantly higher risk of attending special education and lower risk of getting a high track recommendation for secondary school, compared to children with score of 10*.			

## Introduction

The Apgar score is a standardized, widely used tool for the rapid assessment of the neonatal condition during the transition to extrauterine life [1]. While primarily used in the clinical setting immediately after birth, the Apgar score has also been shown to predict both short-term and long-term outcomes. A low 5-min Apgar score is a well-established predictor of neonatal mortality, and has also been associated with neurological disabilities such as cerebral palsy, attention deficit/hyperactivity disorder, and epilepsy even in the era of therapeutic hypothermia treatment for hypoxic ischemic encephalopathy [2–7].

A recent study of Selvaratnam et al. in Australia demonstrated a graded association between the 5-min Apgar score and developmental and educational outcome throughout primary and early secondary school [8]. The most unfavourable outcomes were observed in children with an Apgar score of 0 to 3. Interestingly, even higher Apgar scores of 7, 8, and 9 and generally considered within the normal range were associated with poorer outcomes compared to a score of 10. Other studies have also shown an association between lower Apgar scores and poorer educational outcome [6, 8–11]. However, some uncertainty remains, as other studies have failed to establish a link between low Apgar scores and lower educational achievements or intelligence [12–14]. It is clinically important to gain a better understanding of the possible association between low Apgar scores and educational outcomes, particularly in light of our previous study showing an increasing trend in low 5-min Apgar scores among (near) term singletons in the Netherlands, which may indicate that more children are at risk for adverse long-term outcomes.

This study aims to investigate the association between the full range of the 5-min Apgar scores (from 0 to 10) and primary school performance in a Dutch cohort. Specifically, we focused on the use of special education and track recommendation for secondary school.

## Materials and methods

### Database

For this national population-based cohort study, data were obtained from the Dutch Perinatal Registry (PERINED) and Statistics Netherland’s database. PERINED contains nationwide clinical data on pregnancies, deliveries, and neonatal (re)admissions, covering 97% of all births occurring beyond 22 weeks of gestation [15]. Statistics Netherlands maintains comprehensive records collected by the Dutch government and various public institutions (www.microdata.nl). The linkage between the Dutch Perinatal Registry and Statistics Netherlands’ Personal Record Database was performed at the individual level within a secure research environment. This process, carried out by Statistics Netherlands, utilized maternal date of birth, child’s date of birth, child’s sex, and the four-digit postal code for probabilistic linkage. After linkage, the records were pseudonymised by Statistics Netherlands.

This study included all singletons liveborn between January 1, 2000, and December 31, 2009. Children were excluded if they had a gestational age (GA) of less than 35 + 0 weeks or more than 42 + 6 weeks, had congenital anomalies, lacked a recorded 5-min Apgar score, or died before the age of 14 years.

Approval for use of the data for this study was obtained from PERINED (number 19.43) and Statistics Netherlands (project number 8617). Under Dutch law, no separate ethical approval for studies using national registry data was required.

### Educational outcomes

We studied two educational outcomes at primary school: the use of special education, considered an indicator of lower academic performance, and receiving a high track recommendation for secondary school, reflecting higher academic achievement.

In the Netherlands, formal education begins at the age of four and includes eight years of primary education. There are several types of primary education: mainstream primary education, special education and special *primary* education. Special education is intended for children with severe visual impairments (cluster 1), children with severe hearing impairments, problems with speech and communication (cluster 2), physical or mental retardation and learning difficulties (cluster 3) and children with severe behavioural and/or psychiatric problems (cluster 4). Special *primary* education is intended for children with milder special educational needs, who are struggling in mainstream education, but do not meet the criteria for special education. This form of education is characterized by smaller class sizes and a less distracting environment compared to mainstream education. All children attending special education are annually registered in a national compulsory registry available at Statistics Netherlands.

At the end of primary school – typically around the age of twelve—each child receives a personalized recommendation regarding the most suitable types of secondary education. There are four types of secondary education: special secondary education, preparatory secondary vocational education (in Dutch “VMBO”), senior general secondary education (in Dutch “HAVO”) and pre-university education (in Dutch “VWO”). The personalized recommendation for secondary school is given by the teacher, and takes into account the academic abilities, interests, motivation and study skills of the child. In addition, all children are obliged to complete a nationally standardized attainment test. If a child’s test score is higher than the teacher’s initial recommendation, the personalized recommendation may be upgraded to a higher track. However, when the test score is lower, the original recommendation remains unchanged.

For the purpose of this study, we identified whether a child attended special education at primary school for a period of one year or longer, including both special education and special *primary* education. Additionally, we studied the recommendation for secondary education, focusing specifically on whether a child received a high track recommendation—defined as a recommendation for either senior general secondary education (HAVO) or pre-university secondary education (VWO).

### Covariates

Several maternal, pregnancy and child characteristics were collected. Maternal characteristics included age in years, ethnicity (Western versus non-Western), socio-economic status (SES), and highest level of education. Neighbourhood SES was based on mean household income, education level, and unemployment level at a four-digit zip code level. It was expressed in quantiles, with Q1 being the least and Q5 being the most affluent. The highest achieved maternal education level was obtained from Statistics Netherlands registry and divided into low (primary school or lower vocational education), intermediate (secondary vocational education or senior general secondary education), high (university education or applied science) or unknown (missing).

Pregnancy-related factors included: parity (nulliparous P0, primiparous P1-2, or multiparous P3 +), cephalic presentation, induction of labour, mode of delivery (spontaneous vaginal, instrumental, planned or emergency caesarean section), hypertensive disease during pregnancy, gestational diabetes, level of care (primary or secondary care at onset of labour and delivery). In the Netherlands, the level of maternity care is based on risk selection. Antepartum judged low-risk pregnancies are under surveillance of primary care, whereas high-risk pregnancies are under secondary care. Primary care is provided by community midwives and on small-scale general practitioners, and includes home births, and birth in a birth centre or in a hospital under the care of a midwife. Secondary obstetric care is provided by an obstetrician in a general or tertiary hospital. Neonatal characteristics that were collected included GA at delivery, gender, birth weight in grammes, small for gestational age (SGA) and large for gestational age (LGA). SGA was defined as < 10 percentiles for gestational age and sex according to the Dutch reference curves. LGA was defined as > 90 percentiles [16].

### Statistical analysis

Data were analyzed in the secure environment of Statistics Netherlands. Statistical analysis was conducted with the use of SPSS (version 25.0) and R and R studio using MICE package (version 4.2.3.). We calculated baseline characteristics for the total population and for groups of children based on their 5-min Apgar score (score of 0–3, 4–6, 7, 8, 9, and 10). Categorical data were reported as absolute numbers and percentages and tested with a chi-square test. Continuous variables were reported as mean with standard deviation and tested with a t-test. Maternal education level was missing in 54.9% of the records. To account for this, we included a dummy variable for “unknown maternal education” as a separate variable in our adjustment models. For the remaining covariates, the proportion of missing data was low (< 4%), and these missing values were handled using single imputation. This was applied because the proportion of missing data of co-variates was below 5% and the Little’s Missing Completely At Random (MCAR) test yielded a non-significant result (p-value 0.594), indicating that the missing values were Missing Completely At Random. We tested multicollinearity on the covariates using the Variance Inflation Factor (VIF).

We explored the association between the Apgar score and the two educational outcomes: use of special education and a high track recommendation for secondary school. The proportion of children attending special education was calculated for the different Apgar score groups. Logistic regression was then used to calculate the crude and adjusted odds ratios (cOR and aOR) with 95% confidence intervals (95% CI). The subgroup with Apgar score of 10 was used as the reference group. An adjustment approach was applied to account for potential confounders using three different adjustment models. Adjustment model 1 included maternal age, parity, ethnicity, SES, maternal education level, child’s sex, and year of birth. Adjustment model 2 included all variables from model 1, with the addition of gestational age. Adjustment model 3 included all variables from model 2, plus cephalic position, mode of delivery, SGA, and LGA status to ensure a more comprehensive assessment of influencing factors. All analyses were repeated for the second educational outcome: a high track recommendation for secondary school.

As maternal education is a strong determinant of the educational outcomes of a child [17, 18], we analyzed its role in the association between the 5-min Apgar score and use of special education at primary school for possible interaction. In case of interaction, we did a stratified analysis: for each Apgar score group, we calculated the rate of special education use stratified by maternal education level (low, median, high and unknown maternal education).

In sensitivity analysis, we first restricted the population to term-born only by excluding children born with GA between 35 + 0 and 36 + 7 weeks. Secondly, we restricted the study population to children born between 2000 and 2007, since therapeutic hypothermia for perinatal asphyxia was introduced in the Netherlands in 2008. Finally, we conducted a third sensitivity analysis by performing logistic regression analyses using the Apgar score as a continuous variable, instead of categorizing the lower Apgar scores in groups of 0–3 and 4–6.

## Results

### Study population

**Fig. 1:**
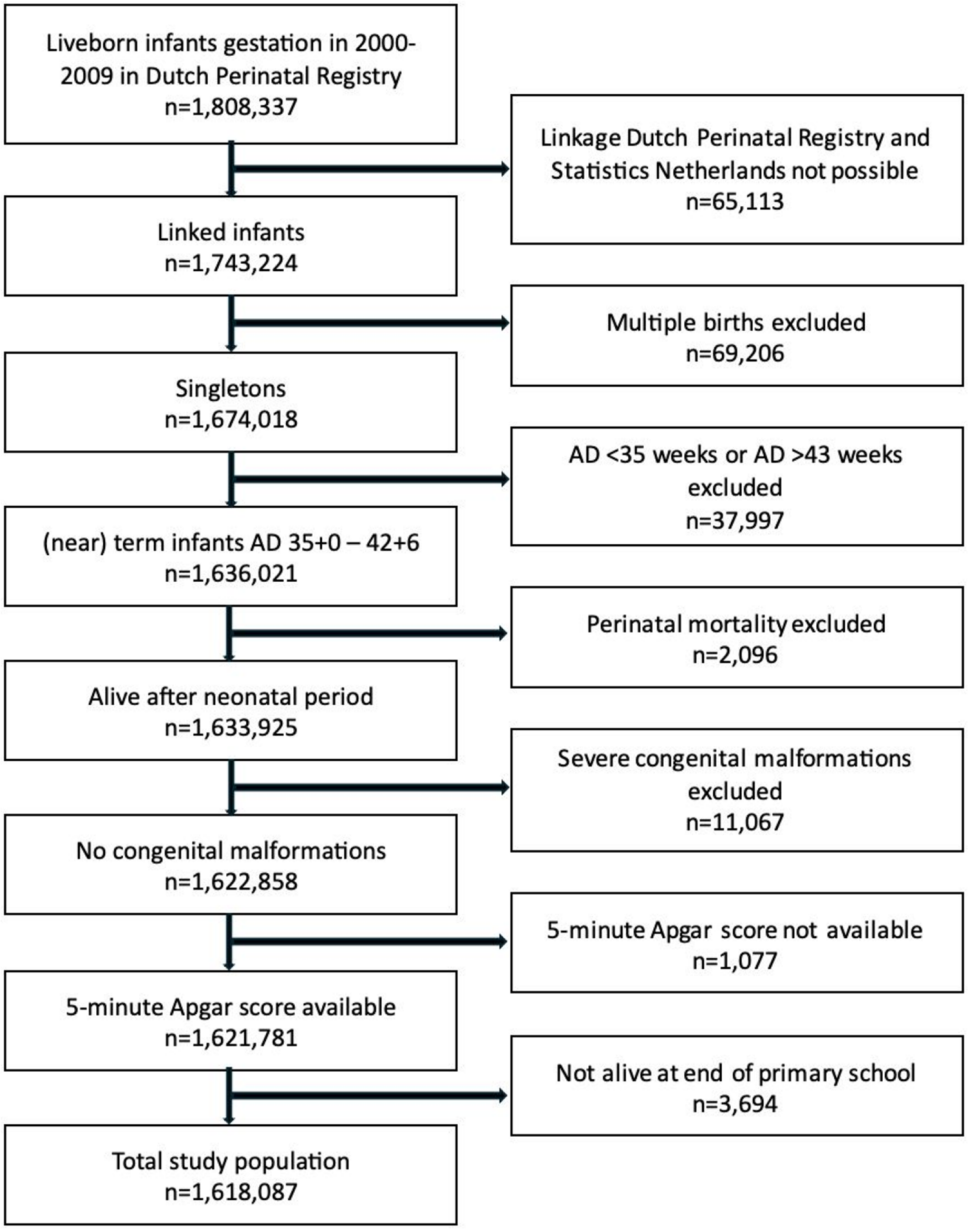
Flowchart study population

In total, 1,808,337 liveborn children were registered in the Dutch Perinatal Registry between 2000 and 2009 (Fig. 1). Linkage with Statistics Netherlands’ Personal Record Database was successful in 96.4%. After exclusion of 69,206 multiple births, a total number of 1,674,018 singletons remained. Of these singletons, 97.7% were born with GA between 35 + 0 and 42 + 6 weeks. We excluded 5,790 children that died before the end of primary school, 11,067 children with congenital malformations and 1,077 children with unknown Apgar score. The final study population included 1,618,087 children.

The main characteristics of the total study population and subgroups of children based on their 5-min Apgar score are presented in Table 1. Supplementary Table 1 shows an extensive version of this Table with additional characteristics. The majority of children had an Apgar score of 10 (76.4%; *n* = 1,236,421). Lower Apgar scores were gradually less frequently observed, with an Apgar score of 9 in 18% of children (*n* = 290,501) and an Apgar score between 0 and 3 in only 0.1% (*n* = 1,493) of children. Children with Apgar score between 0 and 3 were more often born to nulliparous women, women of non-Western origin, and those with low SES or low levels of maternal education. An instrumental vaginal delivery and emergency caesarean section were more frequently performed in the group with Apgar score 0–3. These children were also more likely to have a lower birth weight, male sex, and SGA status.

**Table 1:** Characteristics of the total study population and subgroups of children based on their 5-min Apgar score

	Total	Apgar 0–3	Apgar 4–6	Apgar 7	Apgar 8	Apgar 9	Apgar 10
*n*	1,618,087	1493	11,671	18,591	59,410	290,501	1,236,421
% of study population	100%	0.1%	0.7%	1.1%	3.7%	18.0%	76.4%
Maternal age; *years*	30.5 ± 4.8	30.4 ± 5.2	30.4 ± 5.1	30.3 ± 5.0	30.4 ± 4.9	30.4 ± 4.9	30.5 ± 4.8
Non-Western ethnicity	15.2%	22.0%	19.3%	17.4%	15.7%	14.3%	15.2%
SES, Q1 (least affluent)	18.9%	22.4%	22.5%	21.5%	20.2%	18.5%	18.8%
Low maternal education	8.9%	11.3%	11.2%	10.8%	9.7%	9.2%	8.7%
Nulliparous parity	46.2%	60.3%	64.2%	63.0%	57.8%	50.8%	44.1%
Cephalic presentation	95.2%	93.0%	93.2%	93.7%	94.0%	94.4%	95.5%
Spontaneous vaginal delivery	75.3%	51.6%	45.6%	49.2%	56.1%	66.0%	79.2%
Emergency CS	7.9%	25.0%	23.1%	17.9%	14.7%	10.5%	6.6%
Birth in secondary care	64.1%	76.7%	85.8%	85.8%	81.7%	75.7%	59.9%
Gestational age; *days*	279 ± 10	278 ± 12	279 ± 12	279 ± 12	279 ± 12	279 ± 11	279 ± 10
Male sex	51.2%	56.2%	58.7%	57.6%	56.9%	54.3%	50.1%
Birth weight; *grammes*	3500 ± 518	3380 ± 648	3410 ± 616	3435 ± 598	3488 ± 586	3530 ± 557	3495 ± 502
SGA (< P10)	10.2%	19.6%	17.8%	16.0%	13.3%	11.0%	9.7%
LGA (> P90)	10.9%	11.5%	12.1%	11.7%	12.9%	13.1%	10.3%

Data presented as mean ± standard deviation (SD) or as percentages, as indicated

*CS* caesarean section, *LGA* large for gestational age, *n* number, *Q* quintile, *SES* socioeconomic status, *SGA* small for gestational age

Missing values in the key covariates were low (< 4%) and imputed: maternal age (*n* = 12), parity (*n* = 81), birth weight (*n* = 293), ethnicity (*n* = 11,014), and SES (*n* = 50,728). The level of maternal education was missing in 54.9% of children. A dummy variable was included for the “unknown maternal education” and used in the adjustments as a separate category.

### Educational outcomes

Data on the use of special education in primary school were available for all 1,618,087 children (Table 2). A total of 6.4% (*n* = 102,973) children attended special education. The special education rate was highest among children with the lowest Apgar scores of 0 to 3 (14.3%), and gradually declined to 6.0% in the group of children with the highest score of 10. Compared with an Apgar score of 10, children in lower Apgar score groups all exhibited a significantly higher aOR for the use of special education. The aOR was the highest for children in the lowest Apgar score group of 0 to 3, with fully aOR of 2.38 (95% CI 2.05–2.77). After adjustment for potential confounders the results were comparable (Table 2). Even children with Apgar score of 9 showed a significantly higher aOR of 1.16 (95% CI 1.14–1.19) for the use of special education, compared with an Apgar score of 10.

**Table 2:** The 5-min Apgar score and use of special education at primary school

Apgar score	Number of children	Special education		cOR (95% CI)	aOR #1 (95% CI)	aOR #2 (95% CI)	aOR #3 (95% CI)
		*N*	%				
0–3	1493	214	14.3	2.62 (2.27–3.03)	2.56 (2.22–2.99)	2.55 (2.19–2.96)	2.38 (2.05–2.77)
4–6	11,671	1257	10.8	1.89 (1.78–2.01)	1.80 (1.69–1.91)	1.78 (1.68–1.90)	1.69 (1.59–1.79)
7	18,591	1693	9.1	1.57 (1.49–1.65)	1.49 (1.41–1.57)	1.47 (1.40–1.55)	1.41 (1.34–1.49)
8	59,410	4836	8.1	1.39 (1.35–1.43)	1.33 (1.29–1.37)	1.32 (1.28–1.36)	1.28 (1.25–1.33)
9	290,501	20,842	7.2	1.21 (1.19–1.23)	1.17 (1.15–1.19)	1.17 (1.15–1.19)	1.16 (1.14–1.18)
10	1,236,421	74,131	6.0	1.00 (Reference)	1.00 (Reference)	1.00 (Reference)	1.00 (Reference)
Total	1,618,087	102,973	6.4				

^#^1: adjusted for maternal age, parity, ethnicity, social economic status, maternal education, sex and year of birth; #2: adjusted for #1 including gestational age; #3: adjusted for #2 and SGA, LGA, cephalic position and type of delivery

*aOR* adjusted odds ratio, *cOR* crude odds ratio, *CI* confidence interval, *GA* gestational age, *LGA* large for gestational age, *SGA* small for gestational age

Table 3 shows the results for a high track recommendation for secondary school. A secondary school recommendation was available for 1,534,805 children, representing 94.9% of the total study population. Among these children, 44.7% (*n* = 685,611) received a recommendation for a high track in secondary school. The percentage of a high track recommendation was the lowest in the group of children with the lowest Apgar scores of 0 to 3 (40.7%), and gradually increased to the highest percentage of 45.0% in the group with score of 10. Compared with an Apgar score of 10, all other Apgar score groups showed a significantly lower aOR for a high track recommendation for secondary school. Children with Apgar scores of 0–3 showed the lowest OR with a cOR of 0.84 (95% CI 0.75–0.94) and fully aOR of 0.86 (95% CI 0.76–0.97) for a high track recommendation. If the children with special education were excluded from the analysis, the results were comparable (Table 4). All adjustment variables had a VIF value between 1.0 and 2.3, indicating no multicollinearity.

**Table 3:** The 5-min Apgar score and a high track recommendation for secondary school

Apgar score	Number of children	High track recommendation		cOR (95% CI)	aOR #1 (95% CI)	aOR #2 (95% CI)	aOR #3 (95% CI)
		*N*	%				
0–3	1293	526	40.7	0.84 (0.75–0.94)	0.83 (0.74–0.93)	0.83 (0.74–0.93)	0.86 (0.76–0.97)
4–6	10,747	4383	40.8	0.84 (0.81–0.87)	0.82 (0.79–0.85)	0.82 (0.79–0.85)	0.85 (0.82–0.89)
7	17,350	7329	42.2	0.89 (0.87–0.92)	0.87 (0.85–0.90)	0.87 (0.85–0.90)	0.90 (0.87–0.93)
8	55,742	23,842	42.8	0.91 (0.90–0.93)	0.89 (0.88–0.91)	0.89 (0.88–0.91)	0.91 (0.89–0.92)
9	275,043	120,779	43.9	0.96 (0.95–0.96)	0.95 (0.94–0.96)	0.95 (0.94–0.96)	0.95 (0.94–0.96)
10	1,174,630	528,752	45.0	1.00 (Reference)	1.00 (Reference)	1.00 (Reference)	1.00 (Reference)
Total	1,534,805	685,611	44.7				

^#^1: adjusted for maternal age, parity, ethnicity, social economic status, maternal education, sex and year of birth; #2: adjusted for #1 including gestational age; #3: adjusted for #2 and SGA, LGA, cephalic position and type of delivery

*aOR* adjusted odds ratio, *cOR* crude odds ratio, *CI* confidence interval, *GA* gestational age, *LGA* large for gestational age, *SGA* small for gestational age

**Table 4:** The 5-min Apgar score and high track recommendation for secondary school in the 1, 458,881 children without use of special education at primary school

Apgar score	Number of children	High track recommendation		cOR (95% CI)	aOR #1 (95% CI)	aOR #2 (95% CI)	aOR #3 (95% CI)
		*N*	%				
0–3	1292	524	44.0	0.88 (0.79–0.99)	0.87 (0.77–0.98)	0.87 (0.77–0.98)	0.87 (0.77–0.98)
4–6	9910	4363	44.0	0.88 (0.85–0.92)	0.85 (0.82–0.89)	0.85 (0.92–0.89)	0.85 (0.92–0.89)
7	16,220	7293	45.0	0.92 (0.89–0.95)	0.89 (0.86–0.92)	0.89 (0.86–0.92)	0.89 (0.86–0.92)
8	52,350	23,742	45.4	0.93 (0.92–0.95)	0.91 (0.89–0.92)	0.91 (0.89–0.92)	0.91 (0.89–0.92)
9	2,59,839	120,333	46.3	0.97 (0.96–0.98)	0.96 (0.95–0.97)	0.96 (0.95–0.97)	0.96 (0.95- 0.97)
10	1,119,370	527,144	47.1	1.00 (Reference)	1.00 (Reference)	1.00 (Reference)	1.00 (Reference)
Total	1,458,881	683,399	46.8				

^#^1: adjusted for maternal age, parity, ethnicity, social economic status, maternal education, sex and year of birth; #2: adjusted for #1 including gestational age; #3: adjusted for #2 and SGA, LGA, cephalic position and type of delivery

*aOR* adjusted odds ratio, *cOR* crude odds ratio, *CI* confidence interval, *GA* gestational age, *LGA* large for gestational age, *SGA* small for gestational age

**Fig. 2:**
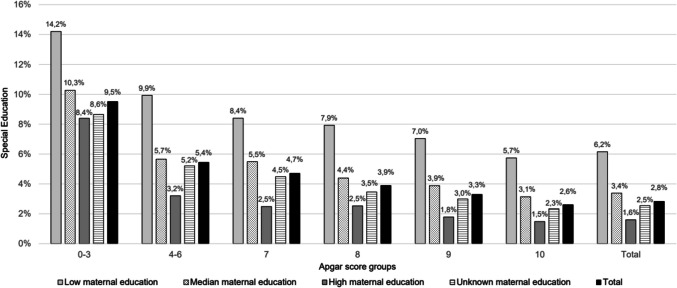
Rate of special education in the Apgar score groups stratified by level of maternal education (low, median, high and unknown maternal education)

There was a significant interaction between Apgar score and maternal education. Figure 2 shows the rates of special education by maternal education level across the different Apgar score groups. All Apgar score groups exhibited a comparable pattern, with the highest rates of special education observed in the low maternal education category and the lowest rates in the high maternal education category. Children from mothers with low maternal education in the low Apgar score group 0–3 showed the highest rate of special education use (14.2%). In addition, Fig. 2 also shows that the unknown maternal education category showed results on special education percentages comparable to those of the median maternal education group.

The first sensitivity analysis in term born children only showed similar results on the use of special of education and a high track recommendation for secondary school (data not shown). The second sensitivity analysis excluding the years when therapeutic hypothermia was introduced also gave similar results (data not shown). The third sensitivity analysis, treating the 5-min Apgar score as a continuous variable without categorization, also demonstrated that higher Apgar scores were significantly associated with a lower risk of requiring special education in primary school and a higher likelihood of receiving a high track recommendation for secondary school (Supplementary Table 2).

## Discussion

In this nationwide study on a population level, we investigated the association between the full range of 5-min Apgar scores and educational outcomes at primary school. The prevalence of special education was more than two times higher in infants with an Apgar score 0–3 compared with the children with an Apgar score of 10. Even children born with an Apgar score of 7, 8 or 9, generally considered within the normal range, had a significantly higher risk of attending special education at primary school. Similarly, a high track recommendation for secondary school was significantly less frequently given to children with lower Apgar scores, compared with children with an Apgar score of 10. Notably, significant and clinically relevant differences were observed across the entire Apgar score range, including children with a high Apgar score of 7,8 or 9. In addition, children of mothers with low education levels showed the highest risks of adverse educational outcomes in each Apgar score group.

The Apgar score was developed to assess a newborn’s condition immediately after birth. Its association with long-term outcomes, as shown in the present study focusing on educational performance at primary school, is intriguing—especially given the score’s simplicity, and its known limitation in interobserver reliability [19]. We believe that the observed association between the Apgar score and educational performance at primary school is particularly concerning in light of our previous study showing a rising trend in low 5-min Apgar scores among (near) term singletons in the Netherlands [20]. Between 2010 and 2019, the proportion of children with Apgar scores < 7 increased significantly from 1.04 to 1.42% (p < 0.001), and those with scores < 4 from 0.17 to 0.19% (p = 0.009). A recent study by Boos and Bührer reported similar results in Germany, with an increasing rate of low 5-min Apgar scores between 2008 and 2022 [21]. Taken together, these findings suggest a potentially growing number of children at risk for long-term adverse educational outcomes and a greater need for special education. In the Netherlands, the number of children requiring special education has already increased in recent years, and nearly one-third of special schools currently have a waiting list [22, 23]. This places additional strain on an already struggling educational system, which is facing rising costs and staff shortages – challenges that are expected to increase even further in the future [24]. Our findings also highlight the importance of further research into the underlying causes of the rising prevalence of low Apgar scores, as well as the strategies to mitigate the risk factors for a low Apgar score.

Despite variations in educational systems across different countries, our findings are consistent with international findings on the association between the Apgar score and educational outcomes. A recent study by Selvaretnam et al., involving 167,126 children in Australia, showed that Apgar scores below 10 were associated with poorer educational outcomes at school grade 3, 5 and 7 (8). Similarly, a study by Tweed et al. in Scotland found an inverse association between Apgar scores and additional support needs (6). In Denmark, children with low Apgar score were less likely to complete upper secondary education and to attend conscription (10). Our findings align with these international studies, by showing that children with Apgar score below 10 are at increased risk of requiring special education in primary school and are less likely to receive a high track recommendation for secondary school.

We analyzed data from a large national cohort, encompassing the full range of 5-min Apgar scores from 0 to 10, and examined both lower academic achievement (measured by the need for special education) and higher academic achievement (assessed as a high track recommendation for secondary school). This study offers a comprehensive perspective on overall educational attainment. While our findings indicate that children with low Apgar scores are at increased risk for poorer educational outcomes, it is equally important to emphasize the positive side: 85% of children with a very low 5-min Apgar score (0–3) do not attend special education during primary school, and nearly 60% receive a high track recommendation for secondary school. These results highlight the potential for resilience and favourable outcomes, even after a difficult start at birth.

### Strengths and limitations

A key strength of this study is the large cohort of more than 1.6 million children in a population based setting. Additionally, the mandatory registration of special education at a national level enhances the validity of our findings. Due to the small number of infants with low Apgar scores, we chose to categorize these in groups (0–3 and 4–6). This may somewhat reduce nuance in our findings of these very low Apgar score categories. Another limitation is the potential for residual confounding, as we were unable to adjust for possible confounders that were not registered in the perinatal registry (PERINED) or during childhood, such as paternal education, maternal smoking, drug use, or body mass index.

In approximately half of the cases, data on individual maternal educational levels were missing in the national registry, necessitating the inclusion of a dummy category (“unknown maternal education”) in our adjustment models. While this approach retains all records and avoids loss of power, it may introduce bias if the reasons for missingness are related to both the Apgar score and the educational outcomes. Interestingly, the “unknown maternal education” category exhibited patterns comparable to those observed in the median maternal education group. This may suggest that the absence of education data may not have introduced significant bias. Possible reasons are that the education information at school level was not yet available at national level or that the education was completed outside the Netherlands. The patterns of outcomes in the “unknown education group” was similar to that of the median education group.

## Conclusion

In conclusion, this study showed in a large Dutch cohort that there is an association between the 5-min Apgar scores and education performance at primary school. Children with lower Apgar scores were more likely to receive special education and were less likely to receive a higher secondary school level recommendation. This pattern is consistent across the entire spectrum of Apgar scores. To determine whether the observed association emerges early in life or evolves over life, future research should focus on longer-term educational achievements, such as the highest level of secondary education completed. Additionally, future research should focus on identifying risk factors associated with low Apgar scores and developing preventive strategies to mitigate these risks.

## Supplementary Information

Below is the link to the electronic supplementary material.

ESM 1 (DOCX 36.5 KB)

## Data Availability

No datasets were generated or analysed during the current study.

## Abbreviations

95% CI: 95% Confidence interval
aOR: Adjusted odds ratio
cOR: Crude odds ratio
GA: Gestational age
LGA: Large for gestational age
MCAR: Missing Completely At Random
SD: Standard deviation
SES: Socio-economic status
SGA: Small for gestational age
VIF: Variance Inflation Factor
