# Supplementary material for: The 5-min Apgar score and primary school performance: a Dutch nationwide cohort study
Source: Eur J Pediatr. 2025 Oct 25;184(11):714. doi: 10.1007/s00431-025-06526-6 (PMC12553574; doi:10.1007/s00431-025-06526-6)
Supplement: Supplementary file 1 — (DOCX 36.5 KB) [file 431_2025_6526_MOESM1_ESM.docx]

**Supplementary Table 1. Characteristics of the total study population and subgroups of children based on their 5-minute Apgar score**

|  | Total population | Apgar  0-3 | Apgar  4-6 | Apgar  7 | Apgar  8 | Apgar  9 | Apgar  10 | P-  value |
| --- | --- | --- | --- | --- | --- | --- | --- | --- |
| N | 1,618,087 | 1,493 | 11,671 | 18,591 | 59,410 | 290,501 | 1,236,421 |  |
| % of total population | - | 0.1% | 0.7% | 1.1% | 3.7% | 18.0% | 76.4% |  |
| Maternal age; *years* | 30.5 ± 4.8 | 30.4 ± 5.2 | 30.4 ± 5.1 | 30.3 ± 5.0 | 30.4 ± 4.9 | 30.4 ± 4.9 | 30.5 ± 4.8 | * |
| Maternal age   - <25 years - 25–29 years - 30–34 years - 35–39 years - >=40 years | 11.5% 29.1% 39.3% 17.6% 2.5% | 13.7% 27.7% 37.2% 17.5% 3.8% | 13.1% 29.1% 37.0% 17.6% 3.2% | 13.0% 29.4% 37.7% 17.1% 2.9% | 12.2% 29.4% 38.1% 17.4% 2.9% | 11.7% 29.4% 38.9% 17.4% 2.6% | 11.4% 28.9% 39.6% 17.6% 2.4% | * |
| Ethnicity   - Western - Non-Western | 84.2% 15.2% | 76.0% 22.0% | 79.6% 19.3% | 81.7% 17.4% | 83.6% 15.7% | 85.1% 14.3% | 84.1% 15.2% | * |
| Social Economic Status   - Q1 (least affluent) - Q2 - Q3 (mean) - Q4 - Q5 (most affluent) - Unknown | 18.9% 19.7% 18.8% 20.3% 19.3% 3.1% | 22.4% 20.4% 17.8% 19.5% 16.3% 3.6% | 22.5% 19.5% 18.3% 18.9% 18.0% 2.8% | 21.5% 19.8% 18.5% 19.5% 17.9% 2.9% | 20.2% 19.9% 18.7% 19.9% 18.1% 3.1% | 18.5% 20.4% 19.5% 20.8% 17.9% 2.9% | 18.8% 19.5% 18.6% 20.2% 19.7% 3.2% | * |
| Maternal education level   - Low - Intermediate - High - Unknown | 8.9% 17.2% 19.0% 54.9% | 11.3% 17.6% 19.2% 51.9% | 11.2% 18.8% 19.2% 50.8% | 10.8% 18.6% 19.0% 51.6% | 9.7% 18.4% 18.9% 53.0% | 9.2% 17.6% 18.7% 54.5% | 8.7% 17.0% 19.1% 55.2% | * |
| Parity   - Nulliparous - Primiparous - Multiparous | 46.2% 36.1% 17.8% | 60.3% 28.0% 11.7% | 64.2% 24.6% 11.2% | 63.0% 25.4% 11.6% | 57.8% 29.0% 13.2% | 50.8% 33.2% 16.0% | 44.1% 37.4% 18.5% | * |
| Cephalic presentation | 95.2% | 93.0% | 93.2% | 93.7% | 94.0% | 94.4% | 95.5% | * |
| Induction of labor | 13.2% | 17.1% | 20.3% | 19.7% | 18.9% | 16.6% | 12.0% | * |
| Mode of delivery   - Spontaneous vaginal - Instrumental vaginal - Planned CS - Emergency CS | 75.3% 11.1% 5.7% 7.9% | 51.6% 16.6% 6.8% 25.0% | 45.6% 25.7% 5.7% 23.1% | 49.2% 27.7% 5.2% 17.9% | 56.1% 23.8% 5.4% 14.7% | 66.0% 17.6% 5.9% 10.5% | 79.2% 8.5% 5.7% 6.6% | * |
| Hypertensive disease | 5.7% | 7.4% | 8.9% | 8.9% | 8.2% | 6.8% | 5.2% | * |
| Gestational diabetes | 1.0% | 1.7% | 1.7% | 1.7% | 1.3% | 1.2% | 0.9% | * |
| Onset birth secondary care | 40.1% | 49.4% | 52.3% | 51.2% | 49.4% | 46.1% | 37.9% | * |
| Delivery in secondary care | 64.1% | 76.7% | 85.8% | 85.8% | 81.7% | 75.7% | 59.9% | * |
| Male sex | 51.2% | 56.2% | 58.7% | 57.6% | 56.9% | 54.3% | 50.1% | * |
| GA; *days* | 279 ± 9.9 | 278 ± 11.9 | 279 ± 11.9 | 279 ± 11.9 | 279 ± 11.5 | 279 ± 10.6 | 279 ± 9.6 | * |
| GA   - 35+0 – 35+6 weeks - 36+0 – 36+6 weeks - 37+0 – 37+6 weeks - 38+0 – 38+6 weeks - 39+0 – 39+6 weeks - 40+0 – 40+6 weeks - 41+0 – 41+6 weeks - 42+0 – 42+6 weeks | 1.2% 2.4% 5.8% 14.6% 24.0% 28.4% 18.6% 5.0% | 3.5% 4.6% 7.4% 13.6% 18.0% 25.7% 20.7% 6.6% | 3.2% 4.4% 7.2% 12.2% 18.9% 24.9% 21.4% 7.7% | 3.1% 4.4% 6.9% 12.7% 19.1% 24.7% 21.2% 7.9% | 2.8% 4.0% 6.7% 13.3% 19.6% 26.0% 20.1% 7.4% | 1.8% 3.0% 5.9% 13.7% 21.9% 27.4% 20.0% 6.4% | 1.0% 2.1% 5.7% 15.0% 24.9% 28.9% 18.1% 4.5% | * |
| Birth weight; *grams* | 3500 ± 518 | 3380 ± 648 | 3410 ± 616 | 3435 ± 598 | 3488 ± 586 | 3530 ± 557 | 3495 ± 502 | * |
| SGA (<P10) | 10.2% | 19.6% | 17.8% | 16.0% | 13.3% | 11.0% | 9.7% | * |
| LGA (>P90) | 10.9% | 11.5% | 12.1% | 11.7% | 12.9% | 13.1% | 10.3% | * |
| Missing value total in parity, maternal age, GA, ethnicity or SES before imputation. | 3.8% | 5.7% | 3.9% | 3.7% | 3.9% | 3.6% | 3.9% | * |

Abbreviations: CS = Caesarean section; GA = gestational age; LGA = large for gestational age; N = number; Q = quintile; SGA = small for gestational age.
* P-value <0.0001

**Supplementary Table 2. Association between the 5-minute Apgar score and special education at primary school and a high track recommendation for secondary school**

|  | **Special education** | | **High track recommendation** | |
| --- | --- | --- | --- | --- |
|  | **OR** | **95% CI** | **OR** | **95% CI** |
| Crude | 0.860 | 0.854 – 0.866 | 1.041 | 1.036 – 1.046 |
| Adjustment model 1 | 0.874 | 0.867 – 0.880 | 1.050 | 1.045 – 1.054 |
| Adjustment model 2 | 0.875 | 0.869 – 0.882 | 1.049 | 1.044 – 1.054 |
| Adjustment model 3 | 0.886 | 0.879 – 0.893 | 1.041 | 1.037 – 1.046 |

Abbreviations: OR = Odds ratio; CI = 95% confidence interval
